# Rolling circle replication requires single-stranded DNA binding protein to avoid termination and production of double-stranded DNA

**DOI:** 10.1093/nar/gku737

**Published:** 2014-08-12

**Authors:** Cosimo Ducani, Giulio Bernardinelli, Björn Högberg

**Affiliations:** Department of Neuroscience, Karolinska Institutet, 171 77 Stockholm, Sweden

## Abstract

In rolling circle replication, a circular template of DNA is replicated as a long single-stranded DNA concatamer that spools off when a strand displacing polymerase traverses the circular template. The current view is that this type of replication can only produce single-stranded DNA, because the only 3′-ends available are the ones being replicated along the circular templates. In contrast to this view, we find that rolling circle replication *in* *vitro* generates large amounts of double stranded DNA and that the production of single-stranded DNA terminates after some time. These properties can be suppressed by adding single-stranded DNA-binding proteins to the reaction. We conclude that a model in which the polymerase switches templates to the already produced single-stranded DNA, with an exponential distribution of template switching, can explain the observed data. From this, we also provide an estimate value of the switching rate constant.

## INTRODUCTION

The mechanism behind rolling circle replication (RCR) and the rolling circle amplification (RCA)-method is thought to be important in viral replication (1,2) for example in human herpes virus 6A (3) and in hepatitis delta virus (4). Variants of RCA have also been extensively used in a number of biological assays and amplification schemes such as padlock-probes (5,6), multiply-primed or displaced-RCA (7,8), the proximity ligation assay (9), which was for example recently used to investigate the role of PIN1 and CtIP interactions during double-stranded DNA break repair (10).

In order for RCR to proceed, the polymerase involved must possess strong processivity and strand displacement characteristics. One such enzyme is phi29 polymerase, from bacteriophage phi29. This polymerase has been important in a lot of the work described above and also for subsequent work in detection of protein–protein or protein–DNA interactions (11), work on human genome amplification and sequencing (8,12), and recently, RNA sequencing *in situ* (13,14) and work on enzymatic production of high quality long oligonucleotides (15). Bacteriophage phi29 from *Bacillus subtilis* contains a linear, double-stranded 20 kb DNA and its replication occurs *via* a protein-primed mechanism. In fact phi29 DNA polymerase, after catalyzing the first covalent bond between the priming protein, called terminal protein (TP), and the first nucleotide, dAMP, synthesizes a short elongation product before dissociating from TP (16). Replication starts at both ends non-simultaneously and the polymerase is associated with a strong processivity and strand displacement activity (17), properties that are also well explained by crystallography data (18). During the elongation single strand binding (SSB) protein 5 is required to protect the displaced strand from nuclease degradation (19), but also greatly stimulates dNTPs incorporation *in* *vitro*, by blocking nonproductive binding of the DNA polymerase to ssDNA (20).

In most of the reports describing displacement replication from circular DNA, one underlying assumption is that the underlying RCA mechanism is robustly producing single-stranded DNA from their circular templates (21,22). As part of our work describing enzymatic production of oligonucleotides (15), we noticed that under certain conditions, we obtained results that were inconsistent with such an assumption. We observed that only by using single-stranded DNA binding protein (in this case, T4 gene-32) during RCA with phi29 polymerase, were we able to consistently get single-stranded products. Here we report on the results following this observation and show that a model in which the polymerase shifts from its circular template to its displaced single-stranded DNA, in a similar fashion suggested to sometimes occur in phi29 viral replication (23,24), can explain an occurrence of large amounts of double-stranded DNA following a RCR process.

## MATERIALS AND METHODS

### RCA of DNA plasmids and digestion of RCA products

We incubated pUC19 plasmid DNA (1 ng/μl, Invitrogen) with nicking endonuclease Nt.BspQI (0.5 U/μl, New England Biolabs (NEB)) in 1× NEB3 buffer for 2 h at 50°C, and then we heat inactivated the enzyme incubating the reaction mixture at 80°C for 20 min. We then amplified the resulting nicked DNA (0.25 ng/μl) by using phi29 DNA polymerase (0.5 U/μl, Fermentas) for 24 h at 30°C in a 1× phi29 reaction buffer (33 mM Tris acetate, 10 mM magnesium acetate, 66 mM potassium acetate, 0.1% (v/v) Tween 20 and 1 mM Dithiothreitol (DTT); Fermentas) containing dNTP mix (1 mM each; Fermentas) and increasing concentration of single stranded binding protein T4 gene 32 (0–100 ng/μl, NEB). We also performed nicking and amplification reactions with pBluescript II SK (+) (with and without T4 gene 32 protein) in the same conditions. We digested the RCA products of plasmid DNA pUC19 and pBluescript II SK (+) using respectively FastDigest MlyI (0.5 U/μl, Fermentas) which recognizes GAGTC(5/5)^∧^ sites, and FastDigest MlsI, which recognizes TGG^∧^CCA sites. We performed the reactions at 37°C, in 1× Fast Digest buffer, for 2 h to ensure a complete digestion. We loaded the digested products and their corresponding undigested RCA products, on 1.5% agarose gel (0.5× Tris-Borate-EDTA buffer (TBE) which is made of 44.5 mM Tris-borate, 1 mM Na_2_EDTA dissolved in deionized water with the addition of etidium bromide (1 ng/μl, Sigma Aldrich)) and we analyzed them by electrophoresis, at 120 V for 2 h. We acquired images by UV trans-illumination (UVITEC) and we analyzed them by the software ImageJ.

### Fluorescence assay

We repeated RCA experiments with pUC19 for measuring dsDNA production by fluorescence. We mixed in a microtiter plate (Nucleon Delta Surface, Thermoscientific) 8 μl of each reaction with 50 μl of a 200× diluted dsDNA specific dye (picogreen, Invitrogen) in 1× TE buffer. We incubated for 5 min in the dark before recording the fluorescence. We set a synergyMX plate reader (Biotek) to an excitation of 480 (20 nm) and an emission of 528 (20 nm). After setting the auto sensitivity on the highest value we collected 50 reads per well from top using a 1000 ms delay after plate movement. All the measurements were normalized by reaction mixtures prepared in triplicate at 4°C and immediately heat inactivated (80°C for 20 min).

### RCA of MOSIC p378 construct

As substrate of the amplification, we used a homemade 378-nt long pseudogene, designed according to the Monoclonal Stoichiometric Oligonucleotide (MOSIC) method recently published (15). 378-nt long pseudogene amplification by RCA in single stranded form can fold hairpin loops containing a BseGI restriction site which allows, after digestion, the release of a 378 nt long single stranded oligonucleotide while the amplification in double stranded tandem repeats provides double stranded DNA fragments of the same length but in base pairs. We circularized the linear 378-nt pseudogene (5 ng/μl) by T4 ligase (0.25 U/μl; Fermentas) in 1× rapid ligation buffer at 22°C for 10 min, followed by an inactivation step at 65°C for 10 min. We nicked the resulting circular pseudogene (1 ng/μl) by Nb.BsrDI and Nt.BspQI (0.5 U/μl, New England Biolabs) in 1× NEB3 buffer at 65°C for 2 h and we stopped the reaction by heating at 80°C for 20 min. We amplified the nicked circular pseudogene (0.1 ng/μl) at 30°C by RCA and T4 gene 32 (25 ng/μl; NEB) stopping the reaction by heat inactivation (80°C, for 20 min) at different time points. We digested RCA products by BseGI restriction enzyme (0.75 U/μl; Fermentas), which recognizes GGATG(2/0)^∧^ sites, incubating in Tango 1× buffer for 24 h at 55°C. We analyzed digested products on 1.5% agarose gel as described before and also on polyacrylamide gel electrophoresis (PAGE) (20% polyacrylamide, 20% formamide, 8 M urea mixed in 1× TBE which is made of 89 mM Tris-borate, 2 mM Na_2_EDTA dissolved in deionized water) at 180 V for 1:15 h. We acquired images by UV trans-illumination (UVITEC) and we analyzed them by the software ImageJ, measuring the intensities of bands corresponding to double stranded digestion products (agarose gel) and the total DNA (denaturing PAGE). Single stranded products were measured as subtraction of dsDNA from the total DNA and all the data collected were plotted on graphs by using GraphPad Prism software.

## RESULTS

### phi29 DNA polymerase rolling circle replication makes dsDNA

**Figure 1.**
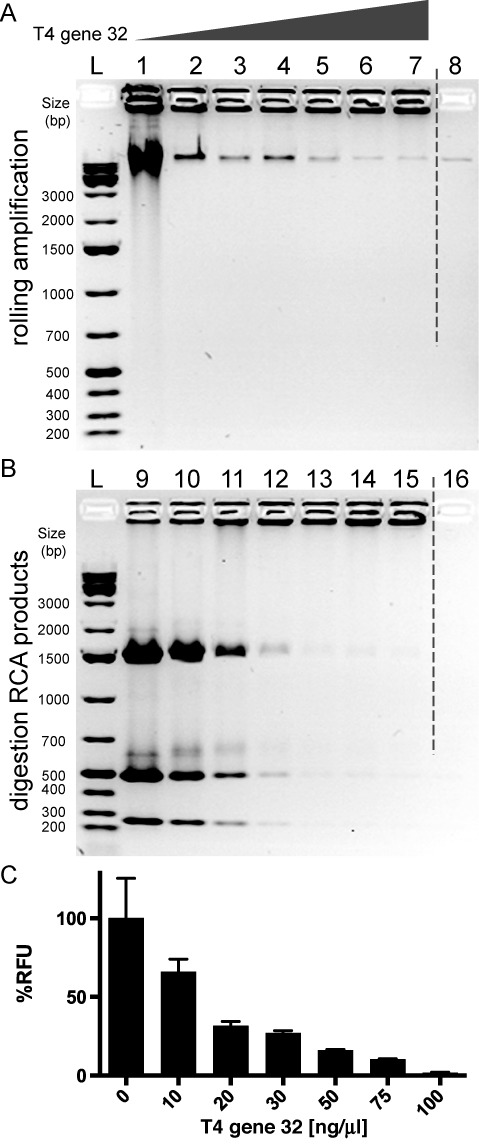
RCA assay of pUC19 DNA plasmid. (**A**) Agarose gel of pUC19 RCA products. Lanes 1–7 RCA performed with increasing concentrations of T4 gene 32 protein (0,10, 20, 30, 50, 75, 100 ng/μl respectively); lane 8 negative control with no phi29 DNA polymerase in the reaction mixture; 1 kb plus DNA ladders (L). (**B**) Agarose gel of MlyI digestion test. RCA products in (A) were digested by MlyI restriction enzyme and the corresponding digestion products (9-16) were run on agarose gel. 1 kb plus DNA ladders (L). (**C**) Picogreen assay of pUC19 RCA. The amplification is expressed in percentage of relative fluorescence units (RFU) and the signal of the amplification product without T4 gene 32 is taken as 100%. Both MlyI digestion and picogreen assay confirm that rolling circle amplification makes mostly double-stranded DNA but they also suggest that T4 gene 32 SSB protein drastically reduces dsDNA production.

**Figure 2.**
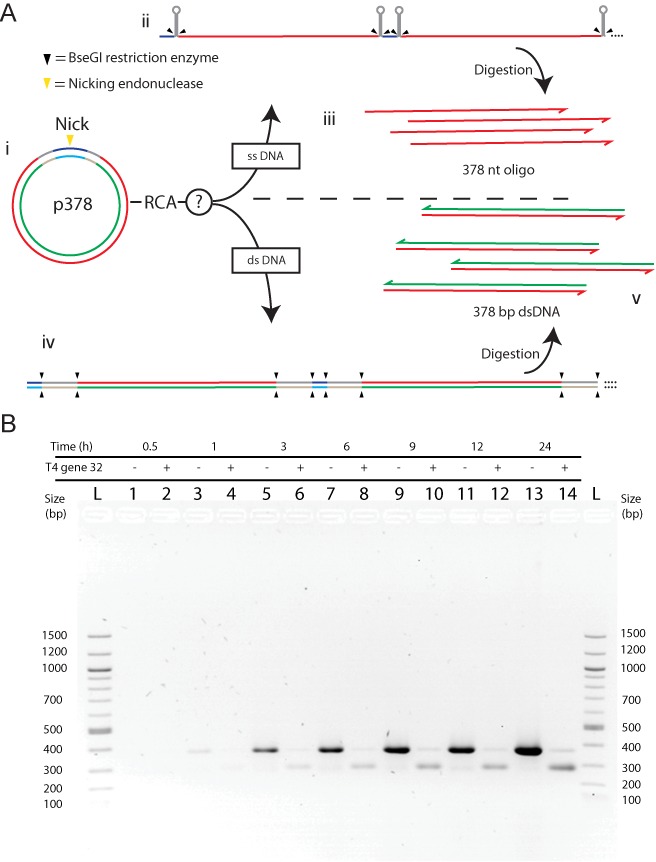
RCA assay in a MOSIC method system. (**A**) Schematic representation of the single-stranded oligonucleotide production by MOSIC method. p378 double-stranded circular nicked DNA (i) is amplified by RCA in two possible ways: single-stranded 378 nt ODN sequence repeated in tandem with hairpin structures in between (ii) and then digested as single-stranded product (iii) or double-stranded DNA repeated in tandem which is digested in double-stranded 378 bp DNA fragments. (**B**) Agarose gel of BseGI digestion products from p378 RCA. RCAs of nicked p378 were stopped at different reaction times from 0.5 to 24 h (lanes 1–14) and the amplifications were performed with (+) and without (−) T4 gene 32 protein. L = 100 bp DNA ladder. The digestion products of RCA performed in absence of T4 gene 32 (odd lanes) correspond to the predicted 378 bp dsDNA which means that phi29 DNA polymerase amplifies p378 mostly in double-stranded form. On the other hand the digestion product of RCA performed with the addition of T4 gene 32 (even lanes) corresponds to the predicted 378 nt ODN as also confirmed from the denaturing PAGE in Supplementary Figure S3.

As first proof of the mechanism we used a pUC19 plasmid as circular template. After a single nicking reaction with Nt.BspQI, we prepared eight amplification reaction mixtures with increasing amount of single strand DNA binding protein, T4 gene 32, and we performed a RCA for 24 h, analyzing the products by agarose gel electrophoresis (Figure 1A). The RCA performed in absence of SSB protein promotes the synthesis of high molecular weight DNA products, plus heavier DNA molecules stuck in the well (Figure 1A, lane 1). By adding increasing concentrations of T4 gene 32 proteins only the heaviest DNA molecules in the well are synthesized while the products with lower mobility gradually disappear (Figure 1A, lanes 2–7). We digested the produced amplicons, by restriction enzyme MlyI, and analyzed the digestion products by agarose electrophoresis (Figure 2B). The digestion of RCA product performed without SSB protein (Figure 1B, lane 9) shows how only the band with higher mobility is completely digested in discrete fragments, while the high molecular weight DNA molecules stuck in the well are not digested. According to a map sequence analysis (Supplementary Figure S1), the length of these products match with a predicted MlyI digestion of a double stranded pUC19, either in a circular form or in a linear tandem-repeat product. The digestion of RCA products provided by increasing amounts of SSB protein shows how these double stranded digestion fragments become fainter and fainter (Figure 1B, lanes 10–15).

To further confirm these results we performed a similar experiment by replacing plasmid DNA pUC19 with pBluescript II SK(+). This time we prepared only two reaction mixtures for our amplification, only one with T4 gene 32 protein. We incubated the RCA products with restriction enzymes and we analyzed the digestion products by electrophoresis. Also in this experiment, we obtained dsDNA digestion fragments only from products of RCA performed without SSB protein (Supplementary Figure S2).

To confirm that the phi29 DNA polymerase amplifies the pUC19 plasmid mostly in double stranded form we also performed a picogreen fluorescence assay evaluating the concentrations of double-stranded DNA (dsDNA) in the RCA reactions. As showed in Figure 1C, 10 ng/μl of SSB protein is sufficient to reduce the amount of dsDNA generated in RCA of pUC19 by around 30%. In the RCA performed with 100 ng/μl of T4 gene 32 the dsDNA signal is close to zero. These data, together with the digestion analysis of the RCA products, suggest that phi29 DNA polymerase amplifies a nicked circular DNA mostly in double stranded form and only the addition of SSB protein T4 gene 32 drastically reduces the amount of dsDNA in the RCA products.

### T4 gene 32 protein promotes ssDNA over dsDNA production

To investigate whether the single strand DNA binding protein not only inhibits the amplification of DNA in double stranded form but also promotes RCA in a single stranded form, we used the MOSIC production method (15). This method is based on producing single stranded oligonucleotides by RCA on a circular nicked DNA and subsequent digestion of hairpin loops between the desired oligonucleotides. As substrate of our RCA we used a 443 bp nicked circular DNA, here on referred to as p378 (the final digested product is 378 nt long), which, after a nicking reaction has been amplified by RCA (Figure 2A(i)). According to our experimental design, a single stranded amplification product would promote the folding of hairpin structures (Figure 2A(ii)) and after digestion of these hairpins, the subsequent release of a single stranded 378 nt oligonucleotide (Figure 2A(iii)), while the amplification of the same DNA if it would occur in double stranded form (Figure 2A(iv)) would release 378 bp dsDNA fragments (Figure 2A(v)).

We performed RCA experiments of the nicked p378 with and without T4 gene 32 protein and we stopped the amplification by heat inactivating at different reaction times (0.5–24 h). Following the amplification we digested the resulting products by BseGI and ran the digested DNA on a native agarose gel (Figure 2B). We observe no high molecular weight bands, indicating that all the RCA products have been completely digested. Further, in all the lanes we noted up to two distinct digestion products: one slower, running at around 400 bp, and one faster running at around 300 bp. When looking at the BseGI digestion products of RCA performed without SSB protein (Figure 2B, odd lanes), we noticed that the main product of digestion was the slower band, and that the intensity of this product increases with the amplification time. The length of this product matches the number of base pairs expected from p378 RCA double stranded DNA product digestion (Figure 2A(v)). On the other hand, by digesting RCA products performed in presence of T4 gene 32 protein (Figure 2B, even lanes) the main product, increasing with the time reaction, is the faster band, which from our MOSIC design can only be single stranded DNA (Figure 2A(iii)). To further confirm this we ran a highly denaturing polyacrylamide gel (Supplementary Figure S3). This gel not only confirms that the band, with higher mobility in the agarose gel, corresponds to the 378 base long oligonucleotide but it also suggests that the total amount of DNA amplified with and without protein is very similar after 24 h and that only the lower affinity of ethidium bromide for single stranded DNA with respect to double stranded DNA makes the single stranded product in the agarose gel appear fainter.

### phi29 makes ssDNA first before turning it into dsDNA

**Figure 3.**
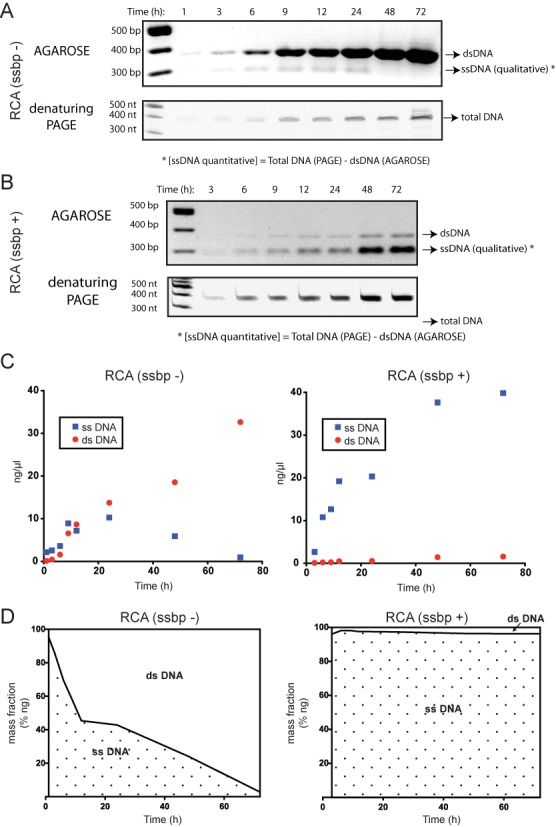
Single and double-stranded DNA production changes in RCA of p378 circular DNA over time. (**A**) BseGI digested RCA products performed without SSB protein over time (1–72 h) loaded on agarose gel (above) and denaturing polyacrilamide gel (below). (**B**) BseGI digested RCA products performed with SSB protein over time (3–72 h) loaded on agarose gel (above) and denaturing polyacrilamide gel (below). (**C**) Plotted concentrations of single and double-stranded RCA products expressed in nanograms per microliter over time. Single-stranded DNA was measured as deduction of the double-stranded DNA band intensities (agarose gels) from the total DNA band intensities (denaturing polyacrilamide gels). Linearity of the band intensities corresponding to the DNA amount range used for our experimental condition was verified (See Supplementary Figure S4). (**D**) Single and double-stranded DNA in RCA over time expressed as mass fraction. In the initial hours of a rolling circle amplification without SSB protein most of the amplicons are in the single-stranded form but then all the DNA is converted into the double-stranded form. The addition of SSB protein T4 gene 32 drastically reduces the conversion of single-stranded DNA into double-stranded form.

Both plasmid DNA and MOSIC construct p378 RCA assays show how phi29 DNA polymerase amplifies a circular DNA mainly in double stranded form and how by adding SSB protein the amplification in single stranded fashion is promoted. This is an unexpected result because the templates for RCA have a single 3′OH each available for starting the amplification. The strand displacement activity of phi29 DNA polymerase is thought to induce the production exclusively of single-stranded, tandem, copies of the nicked strand. To understand the mechanism by which phi29 DNA polymerase makes double stranded DNA from a nicked circular template, and why this production can be prevented by SSB protein T4 gene 32, we decided to measure how the single and double stranded DNA product concentrations change over time in an RCA. To achieve this goal we performed several p378 RCAs, heat inactivating at different reaction times, from 1 to 72 h, and running BseGI digestion products in agarose and denaturing polyacrylamide gels (Figure 3A and B respectively without and with SSB protein). The variation of single and double stranded DNA produced during the RCA was plotted (Figure 3C). In detail, the single stranded DNA was measured as the difference of the double stranded DNA band intensities (agarose gels) from the total DNA band intensities (denaturing PAGE). When performing the reactions without SSB protein, DNA amplicons in single stranded form increase until around 24 h, then decrease drastically almost up to zero after 72 h reaction while dsDNA product concentration continues to increase. In contrast, in the RCAs performed with SSB protein, we observe almost exclusively DNA amplicons in single stranded form (Figure 3C). We also plotted single-stranded DNA (ssDNA) and dsDNA mass ratios over time: it is clear that phi29 DNA polymerase, without SSB protein, first produces single stranded products (95% after 1 h) and gradually turns the majority of it into double stranded form, while in reactions with SSB protein, dsDNA products are <4% even after a 72-h reaction (Figure 3D).

It appears that this mechanism is also active, although probably at a lower degree, in RCR of small circles (see Supplementary Data S5) however, more data would be needed to compare the strand drift rate compared to long template RCR. Also, it should be noted that while we do see dsDNA being produced already at short timescales, the relative impact is more important for long RCR reactions and thus the importance of our observations could be limited for protocols employing shorter reaction times and small circles, like in references (5–6,9,25).

### Model of template switching and dsDNA production

**Figure 4.**
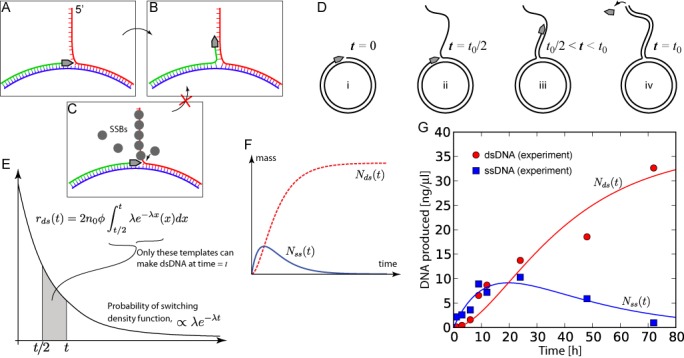
Model of template switching and its implications on ds/ssDNA production. (**A**), (**B**) The template switching event in detail. (A) According to the current view on RCA mechanisms, a polymerase, in grey, produces new DNA (in green) from the circular template (in blue) and continually displaces the 5′-end DNA from the template (in red). We propose that strand switching events, that are randomly distributed with an exponential distribution, converts some of the normal RCA templates into templates for double-stranded DNA production in a mechanism like the one depicted in (B)—switching from the circular template, up to the displaced strand. (**C**) This mechanism can be shut down by single-stranded DNA binding proteins (SSBs) that prevent access to the displaced strand for the polymerase. (**D**) Phases of ssDNA versus dsDNA production of a single template. Initially, all templates are assumed to produce ssDNA in our model. Assuming that a template switching event occurs at time *t*_0_/2, then the polymerase attached to this template will consume the ssDNA that has been produced up to that time while converting it to dsDNA. Assuming that the rate of the polymerase is equal while processing along the single strand, at time *t*_0_ it will stop nucleotide incorporation and fall of the template that is now fully double-stranded. (**E**) Assuming an exponential decay of the templates into dsDNA production with a rate constant of *λ*, the probability density function will determine the instantaneous flipping probability. To get a figure of the current production rate of dsDNA at time *t*, one needs to consider that only the templates that have switched between *t*/2 and *t* will be currently making dsDNA. The rate of ds-production at *t* is thus proportional to an integral over the probability density function between *t*/2 and *t* times the concentration, *n*, and the phi29 rate, *ø*. Once the rate of dsDNA production is known, the rate of ssDNA production can be calculated as the rate of production of all non-switched templates, minus the above ds-DNA rate (because that process consumes already produced ssDNA). Integrating (Supplementary Note S1) the rates, yields expressions for the total mass of produced dsDNA, *Nds*(*t*), and total mass of produced ssDNA, *Nss*(*t*). The expressions are plotted in (**F**) using arbitrary units. (**G**) Quantitative fit of the data in Figure 3C to the theoretical curves (*Nds*(*t*) and *Nss*(*t*) not fitted independently with respect to each other) reveals a close resemblance to the measured data and our model. From this we estimate the rate constant *λ* to be 1.95 ± 0.25 × 10^−5^ events per second per template for the switching process.

From the above results, it appears that dsDNA is produced by an elongation of the polymerase strand along the previously produced ssDNA. We hypothesize that this behavior can be explained by a strand-switching mechanism in which the polymerase drifts, away from its circular template, and settles on the nearby displaced strand at the displacement fork (Figure 4A and B). It appears, that at a high enough concentration of SSB protein, this mechanism is stopped by blocking the displaced strand from the strand switch (Figure 4C). If this mechanism is true, then the polymerase would, in the absence of SSB protein, effectively consume all the displaced ssDNA that was previously produced from that template, and instead turning it into a double-stranded product (Figure 4D). Another possible mechanism could be that the polymerase double-backs onto its own strand, forming a hairpin. Although we cannot rule this mechanism out, we believe, line with previous findings about replication anomalies (23,24), that a strand drift mechanism is the more plausible explanation.

To model this behavior we assume an exponential decay of the templates that originally were producing ssDNA by strand displacement (referred to as *ssDNA templates* below), decaying into templates where the polymerases have switched and starts producing dsDNA by elongation along the previously displaced ssDNA (referred to as *dsDNA templates* below). If the template switching follows an exponential distribution, the concentration of switched templates *n_sw_* at time *t*, after reaction initiation should follow this cumulative distribution function:

\begin{equation*} n_{{\rm sw}} (t) = n_0 (1 - e^{ - \lambda {\rm t}} ) \end{equation*}

where *n*_0_ is the initial concentration of templates. In our model, because the production of dsDNA consumes already produced ssDNA, the instantaneous rate of ssDNA production follows:

\begin{eqnarray*} &&{\rm rate}\,{\rm of}\,{\rm ssDNA}\,{\rm production} = \\ &&{\rm normal}\,{\rm production} - {\rm rate}\,{\rm of}\,{\rm dsDNA}\,{\rm production} \end{eqnarray*}

Thus, to get the rate of ssDNA production, and consequently derive an expression for the total amount of ssDNA at time *t*, we need to first calculate the rate of dsDNA production. In order to accurately quantitate the instantaneous dsDNA production rate we need to consider that for all dsDNA templates, the reaction will stop at some point because the previously displaced ssDNA runs out, see Figure 4D(iv). Thus, the dsDNA production rate, is not simply the number of dsDNA templates (*n*_sw_(*t*)) times the rate of the polymerase, but in fact a term that counts only the number of dsDNA templates that *currently* are making dsDNA, like in Figure 4D(iii). Thus, to evaluate the actual number of templates that can be producing dsDNA, we need to consider only those templates that have switched and have not yet reached termination. To get the amount of switching per unit time, we take the derivative of the cumulative distribution function to get a probability density function that is proportional to $\lambda e^{ - \lambda t}$, see Figure 4E. If a template is terminated, like in Figure 4D(iv), at time *t*, then we can assume it underwent switching at time = *t*/2. This is in turn based on the assumption that the polymerase rate is the same when making ssDNA by strand displacement, as it is when making dsDNA during elongation after switching. That is, if a polymerase has made one long piece of dsDNA, we can assume that it spent half the time making one strand by strand displacement, and the other half of the time filling it out to double-stranded form after a template switching event.

This discussion leads to the fact that to get the dsDNA production rate at time *t*, *r*_ds_(*t*), we need to take the integral over the probability density function (switching rate) between *t* and *t*/2, and multiply that with the rate of the polymerase, the initial concentration and a factor of 2. The doubling factor takes into account that for each nucleotide incorporated, we actually get one base-pair of dsDNA in the final product, so two nucleotides that will appear as dsDNA in our final measurements. From the dsDNA production rate, we can calculate the ssDNA production rate and finally, by integrating, obtain the final expressions for the total concentration *N*, of the two forms of DNA at a given time point *t*:

\begin{equation*} N_{{\rm ds}} (t) = 2n_0 \phi \frac{1}{\lambda }\left( {e^{ - \lambda t} - 2e^{ - \frac{\lambda }{2}t} + 1} \right) \end{equation*}

\begin{equation*} N_{{\rm ss}} (t) = 2n_0 \phi \frac{1}{\lambda }\left( {e^{ - \frac{\lambda }{2}t} - e^{\lambda t} } \right) \end{equation*}

These functions are plotted with arbitrary units in Figure 4F. For a more detailed derivation of these results see Supplementary Note S1. We used a Levenberg–Marquardt algorithm through a python script (Supplementary Figure S6) to obtain a fit to our experimental data for the development of DNA concentrations over time shown in Figure 3C. The result of the comparison between the theoretical model and the experimental data is shown in Figure 4G. Note that the fitting was done based only on the ssDNA data points, and the plotted curves for both *N*_ds_ and *N*_ss_ in Figure 4G used the same fitted parameters for *λ* and 2*n*_0_*ϕ* obtained for this fit. Despite this, the curve for *N*_ds_ closely follows the data points for dsDNA indicating that our model is able to closely describe the observed behavior.

The value obtained for the rate constant through fitting to our was *λ* = 1.95 × 10^−5^ events per second per template, with a standard deviation of 0.25 × 10^−5^. This is equivalent to a half-life of 9.9 h of the ssDNA templates before switching.

## DISCUSSION

We clearly show for the first time, experimental evidence that phi29 DNA polymerase catalyzes the formation of large quantities of double stranded products in RCR. We also demonstrate that this double-strand production can be inhibited by adding a single stranded DNA binding protein during the amplification reaction.

The model we introduce implies that in order for an RCR mechanism to work as hypothesized previously, the displaced strands need to be blocked to prevent template drift of the polymerase. If the concentration of the blocking agent (SSB protein in our case) becomes too low, then the replication will eventually stop (reach the plateau in Figure 4F), regardless of an abundant access to nucleotides. This problem of apparent premature RCA termination is a well-known problem when RCA is used for ssDNA amplification (see for example (25,26)) and our model provides a plausible explanation of this effect. It is evident that, although this termination effect can only be observed after a relatively long time, a considerable fraction of the products will be double-stranded already after a few hours. For this reason our findings will definitively be crucial to improve technologies based on enzymatic amplification of DNA in single stranded form, such as self-assembling nanoarray genome sequencing (12), RNA sequencing *in situ* (14), enzymatic oligonucleotide production (15) and RCA based detection assays (5,6).

In addition, our results imply that other types of displacement amplification mechanisms, such as mitochondrial genome replication, might also be susceptible to similar problems. In fact, template switching-like phenomena have been implicated in relation to other systems such as in Pfu and *Escherichia coli* polymerase III replication (27,28). It thus seems reasonable to assume that many replication systems could be reliant on mechanisms that are in place solely to prevent polymerase template switching events like the ones we report here. Of course, these are speculative assumptions and further studies will have to be conducted to conclude whether our findings can have such implications.

## SUPPLEMENTARY DATA

Supplementary Data are available at NAR Online.

SUPPLEMENTARY DATA
