# Supplementary material for: Rolling circle replication requires single-stranded DNA binding protein to avoid termination and production of double-stranded DNA
Source: Nucleic Acids Res. 2014 Aug 12;42(16):10596–604. doi: 10.1093/nar/gku737 (PMC4176320; doi:10.1093/nar/gku737)
Supplement: SUPPLEMENTARY DATA [file supp_42_16_10596__index.html]

Rolling circle replication requires single-stranded DNA binding protein to avoid termination and production of double-stranded DNA — Rolling circle replication requires single-stranded DNA binding protein to avoid termination and production of double-stranded DNA — SUPPLEMENTARY DATA 

# Rolling circle replication requires single-stranded DNA binding protein to avoid termination and production of double-stranded DNA

## SUPPLEMENTARY DATA

**Files in this Data Supplement:**

- SUPPLEMENTARY DATA
