# Supplementary material for: Rolling circle replication requires single-stranded DNA binding protein to avoid termination and production of double-stranded DNA
Source: Nucleic Acids Res. 2014 Aug 12;42(16):10596–604. doi: 10.1093/nar/gku737 (PMC4176320; doi:10.1093/nar/gku737)
Supplement: SUPPLEMENTARY DATA [file supp_gku737_nar-01502-f-2014-File006.pdf]

Rolling circle replication requires single-stranded DNA binding protein to avoid termination and production of double-stranded DNA

Cosimo Ducani, Giulio Bernardinelli and Björn Högberg

Supplementary information

Figure S1: MlyI restriction analysis
Figure S2: pBluescript RCA digestion
Figure S3: PAGE of BseGI digested product of RCA from p378
Figure S4: Linearity of gel measurements
Figure S5: RCA performed using a small circle
Figure S6: Method and output of model fitting to the data.

Note S1: Detailed derivation of analytical functions for ss/ds-DNA production.

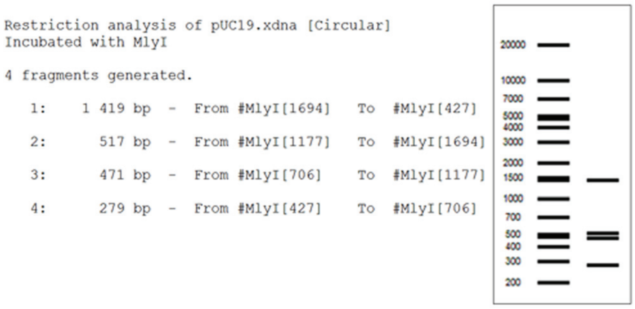

Figure S1. MlyI restriction analysis. Prediction of generated fragments from MlyI digestion of pUC19 plasmid in the circular form.

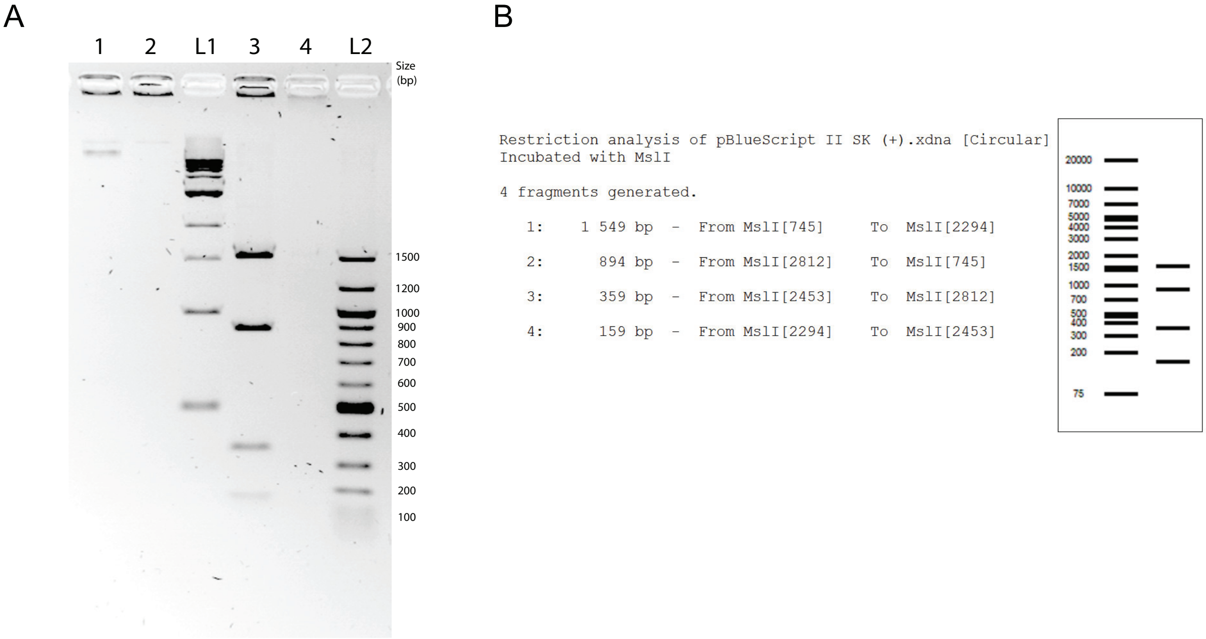

Figure S2. RCA assay of pBluescript II SK(+). (A) RCA of nicked pBluescriptII SK(+) without (1a) and with (1b) T4 gene 32 protein (0.1 µg/µL) and corresponding MslI digestion products run in agarose gel. Only the digestion of RCA products amplified without T4 gene 32 provides double stranded DNA fragments. (B) MslI restriction analysis. Prediction of generated fragments from MslI digestion of pBluescriptII SK(+) plasmid in the circular form.

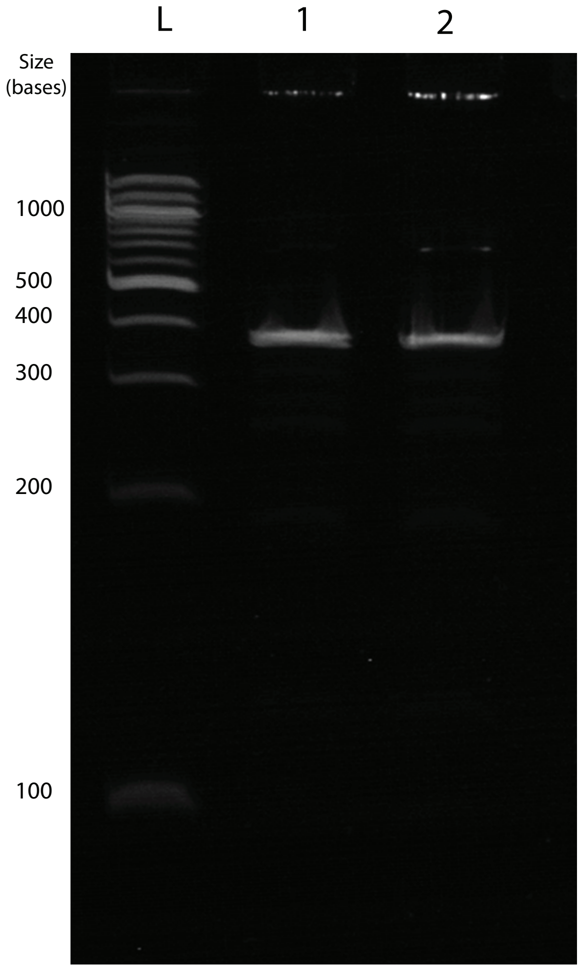

Figure S3: Denaturing PAGE of BseGI digested product of RCA from p378. BseGI digestion of p378 RCA products without (lane 1) and with (lane 2) T4 gene 32 protein.

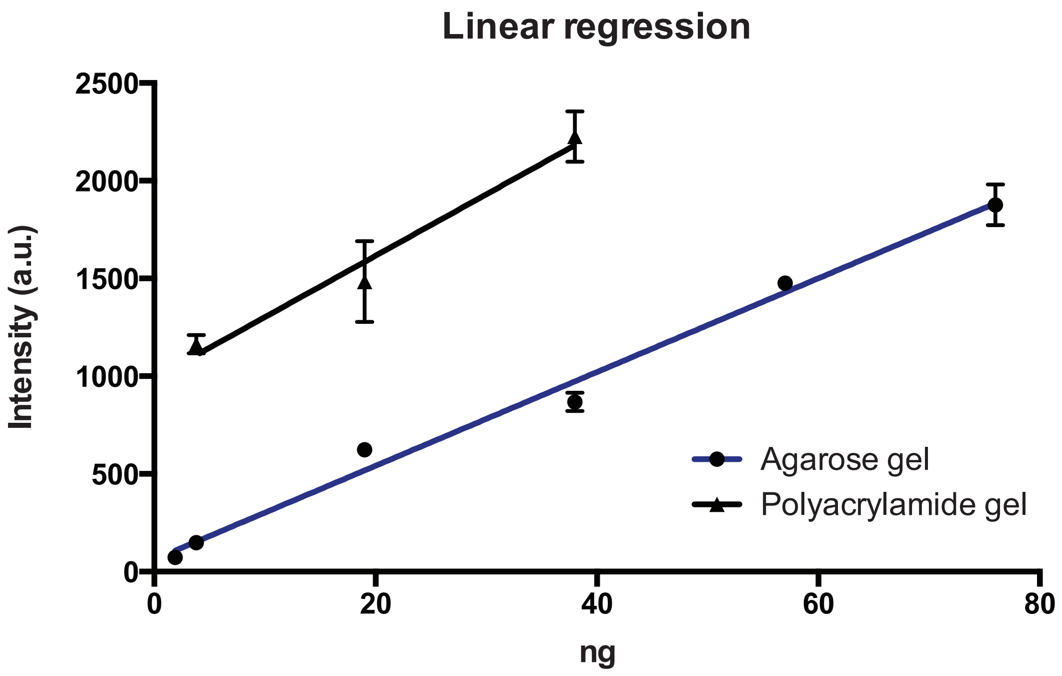

Figure S4. Evaluation of band intensity linearity in electrophoresis experiments.

We tested the linearity of the signal intensity for our gel measurement system for both agarose and polyacrylamide gel electrophoresis by plotting standard curves of known samples for the regions of interest used in the paper. For both agarose gel (1.5%, 0.5x TBE, EtBr staining, ran for 75 min at 180V) and polyacrylamide gel (10%, 8M Urea, 20% formammide, 1x TBE, Sybr gold staining, 75 min at 180V) we measured in triplicate known amount of Quickload 100 bp ladder (NEB). We collected all the data using ImageQuant LAS 4000 imager (GE healthcare) and quantified the density of the 400 bp band using Gel Analyzer Software (http://www.gelanalyzer.com). We calculated a linear regression fit using GraphPad Prism. Within the range of DNA amount detected in the RCA assays performed experiment, we experienced a linearity of the recorded information. The obtained r^2 for single stranded, denaturing polyacrylamide gel, and double stranded DNA, native agarose gel, were, respectively, 0.9858 (Y = 23.98 * X + 62.23) and 0.9606 (Y = 73.06 * X + 932.0).

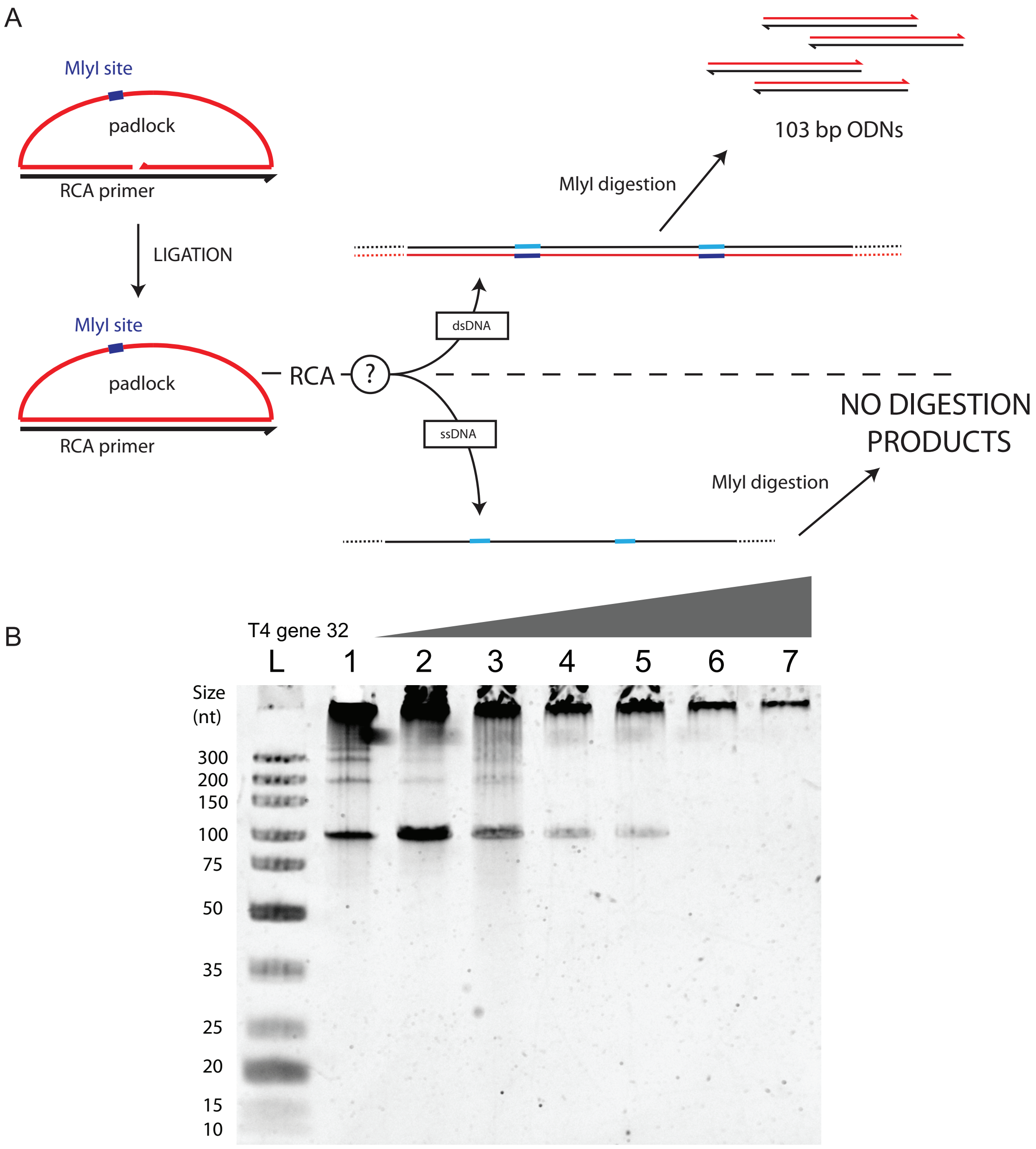

Figure S5. RCA performed using a small circle. (**A**) Schematic representation of RCA experiment performed on a small circle: a 103 nt oligonucleotide (padlock) after phosphorylation in 5' end, was annealed to a shorter oligo (27 nt). The padlock contains a restriction site for MlyI. After ligation (T4 ligase 0.5 U/µl for 10 min at 22 °C) we performed a RCA using the shorter oliog as the primer. The amplification of the padlock in double stranded form (top of the figure) provides active Mly restriction sites, while amplification in ssDNA cannot be digested in 103 nt fragments. Padlock sequence: 5-GTATTCAGTGAATTCCGTTAGTTCGTTATTAACGTAGATTGAGTCTCTTCCCAACGTCCTGAATGGTATAAGCGCCAGTTCTTAAAATCAGTAAAGGCCCGCT-3; RCA primer sequence: 5-ATTCACTGAATACAGCGGGCCTTTACT-3. (**B**) MlyI digestion of padlock RCA products: after priming of the padlock to the RCA primer (95 °C for 1 min and 1 hr cooling at R.T.) in a similar way as before, we performed the RCA (30 °C for 40 hr) with increasing amount of T4 gene 32 protein (0, 25, 50, 100, 250, 500 ng/µl) and we loaded the RCA products on 20% denaturing polyacrylamide gel (20% formamide, 8M urea). We ran the gel for 1:00 hr at 180V. In absence or at low concentration of SSB protein, the reaction contains dsDNA products.

Script:

```
import scipy as sp
import numpy as np
import scipy.optimize as opt

# the time-points in seconds
t=np.array([
3600,
10800,
21600,
32400,
43200,
86400,
172800,
259200,
])

# the amount of ssDNA in ng/ul
data_ss=np.array([
2.16,
2.57,
3.60,
8.90,
7.20,
10.29,
5.89,
0.95,
])

def N_ss(t, c, lamb):
    return c*(sp.exp(-0.5*t*lamb)-sp.exp(-1.0*t*lamb))/lamb

init_const=1
init_lambd=1.8e-5


popt, pcov = opt.curve_fit(N_ss, t, data_ss, p0=np.array([init_const, init_lambd]))

print 'The optimal values are:'
print popt
print 'The covariance of the values are:'
print pcov
print 'The standard deviation errors on the parameters is:'
print np.sqrt(np.diag(pcov))
```

Result of fit:

```
In [16]: run fitting.py
The optimal values are:
[  7.13777626e-04   1.95153775e-05]
The covariance of the values are:
[[  1.20952685e-08   2.23492732e-10]
 [  2.23492732e-10   6.41438993e-12]]
The standard deviation errors on the parameters is:
[  1.09978491e-04   2.53266459e-06]
```

Figure S6. Method and output of model fitting to the data.

The data points for the ssDNA yield were entered into a python script that implemented the $N_{ss}(t)$ as a function, `N_ss`, and then used the `optimize.curve_fit` function of the module SciPy. This function uses a Levenberg-Marquardt algorithm to do a non-linear least square fitting of the data points to the entered function. The output of the script is shown on the right.

Supplementary Note S1
Modelling of DNA production due to strand switching.

Assuming that the concentration of templates is initially n_0 and the switching events (as outlined in fig. 4 A and B) occur randomly following an exponential distribution. The amount of templates that has switched at time t will thus be, $n_{sw}(t)$:

$$n_{sw}(t) = n_0(1 - e^{-\lambda t}) \tag{1}$$

where λ denotes the rate constant of the exponential process. The rate of switching, *i.e.* the probability density function is given by:

$$P_{sw}(t) = \frac{d}{dt} n_{sw}(t) = \lambda e^{-\lambda t} \tag{2}$$

The amount of templates that are producing double-stranded DNA at a certain point in time is *not* directly proportional to the number of switched templates given by (1). It is important to note that because the polymerase is assumed to stop dsDNA production after making the entire tail double-stranded, like in (d) in the figure below. We assume that the polymerase rate is the same when making displaced single-strand, like in (b), as when filling out the tail and making it double-stranded, like in (c). Because of this hypothesized behaviour it takes the same amount of time to convert the tail to double-stranded DNA as it took to create the tail in the first place. Lets assume that a template has reached this stopping point at time t_0, like in (d). Then that template must have switched at time $t_0/2$, like in (b). Thus, the *only* templates that are making double-stranded DNA at time t_0 are the templates that have switched after $t_0/2$. Therefore, assuming the rate of the

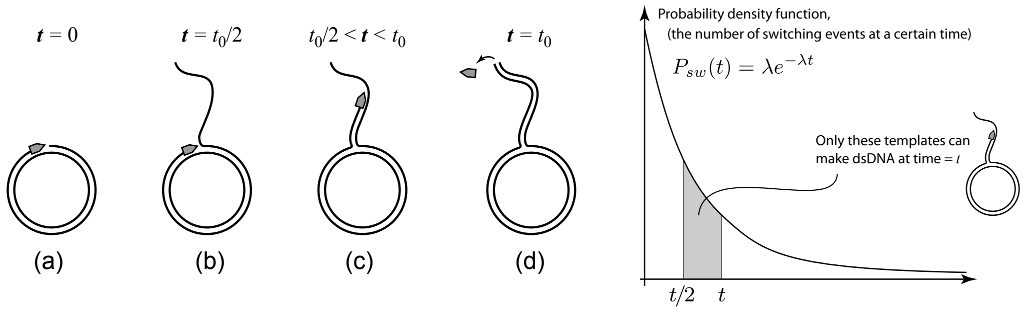

polymerase is ϕ, we get that the instantaneous rate of double stranded production, $r_{ds}(t)$ is given by:

$$r_{ds}(t) = 2n_0\phi \int_{t/2}^{t} P_{sw}(x)dx \tag{3}$$

The 2 in the above equation is taking into account that for each nucleotide incorporated, we actually get one base-pair of dsDNA in the final product, so two nucleotides that will appear as dsDNA in our final measurements. We calculate the integral to get:

$$r_{ds}(t) = 2n_0\phi \int_{t/2}^{t} \lambda e^{-\lambda x}(x)dx = n_0\phi \left[-e^{-\lambda x}\right]_{t/2}^{t} = n_0\phi(e^{-\frac{\lambda}{2}t} - e^{-\lambda t}) \tag{4}$$

To get the amount, in numbers of nucleotides, of produced double-stranded DNA at time t, $N_{ds}(t)$, we take the integral of the rate-equation (4):

$$N_{ds}(t) = 2\int r_{ds}(t) = 2\frac{n_0\phi}{\lambda}\left(e^{-\lambda t} - 2e^{-\frac{\lambda}{2}t} + C_1\right) \tag{5}$$

Ignoring the trace amount of initial template, the total dsDNA should be 0 at $t = 0$, which gives $C_1 = 1$ in expression (5).

The base rate of single-stranded DNA production can be assumed to be proportional to the number of non-switched templates, $n_{ns}(t)$, which relates to equation (1) as:

$$n_{ns}(t) = n_0 - n_{sw}(t) = n_0 - n_0(1 - e^{-\lambda t}) = n_0 e^{-\lambda t} \tag{6}$$

However, there is another term that one needs to consider: All the templates that are currently making double-stranded DNA, as in (c), are *consuming* single-stranded DNA. Thus, the rate of ssDNA production, $r_{ss}(t)$, is the polymerase rate, ϕ, times the number of non-switched templates, given by (6), *minus* the current rate of double-stranded DNA production, given by (4):

$$r_{ss}(t) = n_0 e^{-\lambda t} - r_{ds}(t) \tag{7}$$

$$r_{ss}(t) = n_0\phi\left(e^{-\lambda t} - e^{-\frac{\lambda}{2}t} + e^{-\lambda t}\right) = n_0\phi\left(2e^{-\lambda t} - e^{-\frac{\lambda}{2}t}\right) \tag{8}$$

The total amount, in nucleotides, $N_{ss}(t)$, is given by the integral of the rate (no multiplier this time since the rate is for single-strand production):

$$N_{ss}(t) = \int r_{ss}(t) = \frac{n_0\phi}{\lambda}\left(2e^{-\frac{\lambda}{2}t} - 2e^{-\lambda t} + C_2\right) \tag{9}$$

The boundary condition that this function is zero at time zero (no ssDNA produced from the start) gives $C_2 = 0$.

So we have for the amounts (in nucleotides/mass) for double-stranded vs. single-stranded DNA at time t:

$$N_{ds}(t) = 2n_0\phi\frac{1}{\lambda}\left(e^{-\lambda t} - 2e^{-\frac{\lambda}{2}t} + 1\right) \tag{10a}$$

$$N_{ss}(t) = 2n_0\phi\frac{1}{\lambda}\left(e^{-\frac{\lambda}{2}t} - e^{-\lambda t}\right) \tag{10b}$$

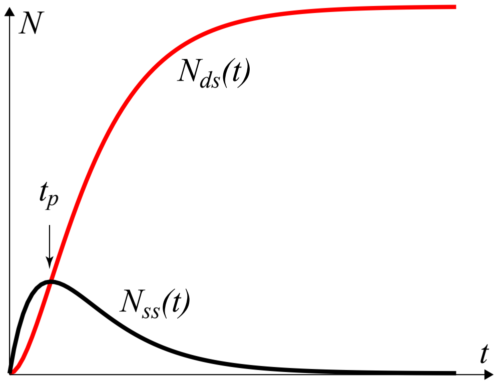

The peak in the single-stranded DNA amount can be experimentally verified. If we call the peak-time when this maxima occurs t_p, we know that the rate of ssDNA production, $r_{ss}(t)$ from (8), is zero at this point, so:

$$\begin{aligned} r_{ss}(t_p) = 0 \;\Rightarrow\; n\phi\left(2e^{-\lambda t_p} - e^{-\frac{\lambda}{2}t_p}\right) &= 0 \\ 2e^{-\lambda t_p} &= e^{-\frac{\lambda}{2}t_p} \\ \ln 2 - \lambda t_p &= \frac{\lambda}{2}t_p \end{aligned}$$

which finally gives the time, t_p, of the peak in terms of the rate constant:

$$\boxed{t_p = \frac{2 \ln 2}{\lambda}} \tag{11}$$

Using our fit of the single-stranded data (Fig. 4 in the paper, and Figure S5) we get that the λ that best describes the data is:

$$\lambda \approx 1.95 \pm 0.25 \cdot 10^{-5} \text{ s}^{-1}$$

giving a peak time of maximum amount of ssDNA at

$$t_p \approx 19.7 \text{ hrs}$$

and a half-life of the non-switched templates of

$$t_{1/2} = \frac{\ln 2}{\lambda} \approx 9.9 \text{ hrs}$$
